# Migraine patients in Germany - need for medical recognition and new preventive treatments: results from the PANORAMA survey

**DOI:** 10.1186/s10194-021-01316-5

**Published:** 2021-09-09

**Authors:** M. Koch, Z. Katsarava, C. Baufeld, K. Schuh, A. Gendolla, A. Straube, W. von Pannwitz, W. E. Hofmann, S. Ortler

**Affiliations:** 1 Novartis Pharma GmbH, Roonstr. 25, 90429 Nuremberg, Germany; 2 Christian Hospital Unna, Unna, Germany; 3 University of Duisburg-Essen, Essen, Germany; 4 EVEX Medical Corporation, Tbilisi, Republic of Georgia; 5 IM Sechenov First Moscow State Medical University (Sechenov University), Moscow, Russian Federation; 6 Essen, Germany; 7 Department of Neurology, Ludwig-Maximilians University, Munich, Germany; 8 Berlin, Germany; 9 Aschaffenburg, Germany

**Keywords:** Migraine, Healthcare systems, Headache, Patient care, Preventive treatment

## Abstract

**Background:**

Migraine is a primary headache disorder characterized by recurrent attacks that may have a significant impact on patients’ daily life. Treatment options must often be re-evaluated in light of efficacy, tolerability and compliance issues. Few data on commonly applied treatment algorithms and treatment failures have existed in Germany in 2017/2018. The PANORAMA survey was designed to explore and characterize the migraine healthcare landscape and to demonstrate the medical treatment need at that time in Germany.

**Methods:**

Three different questionnaires were used to assess the profile of the 119 participating centers, characterize migraine patients at centers and evaluate qualitative aspects of the current migraine healthcare situation from a physician´s professional perspective. Data were analyzed as observed and summarized by descriptive statistics.

**Results:**

The results demonstrate that once referred to a migraine specialist, the majority of patients continue to be treated at a specialist. At specialized centers, 41.6 % of migraine patients receive prophylactic treatment. 45.4 % of prophylactic treatments are initiated with a beta-blocker and 28.1 % with an anti-epileptic. Pivotal factors to initiate prophylactic treatment are migraine attack frequency and intensity (58.0 %). Treatment decisions are largely based on prior / concomitant diseases and physical constitution of the patient (52.1 %). Following an inadequate treatment, most patients either switch substance class or discontinue prophylactic treatment.

**Conclusions:**

PANORAMA gives a comprehensive overview of the migraine healthcare landscape in Germany in 2017/2018, elucidates a lack of common treatment algorithms and reveals a high demand for defined therapy strategies and new prophylactic treatment going forwards.

**Supplementary Information:**

The online version contains supplementary material available at 10.1186/s10194-021-01316-5.

## Main text

### Background

Migraine is a neurological disorder characterized by recurrent attacks of moderate to severe headache with throbbing, pulsatile pain, often occurring unilaterally, and associated with nausea, vomiting and sensitivity to light, sound and sometimes smell. Frequency and intensity of migraine attacks may have a significant impact on patients’ quality of life (QoL) and daily activities, resulting in significant burden of disease [1–3]. Most patients suffering from migraine first present to a primary care physician, before being referred to a headache specialist [4, 5]. Physicians have different treatment options in migraine therapy: acute and preventive migraine medications, as well as non-pharmacological migraine treatment options, which are often used in combination. Most of the earlier established prophylactic pharmacological treatment options have not been developed primarily for the migraine indication. Consequently, they are lacking target specificity and can have significant side effects and possible interactions with other medications for a long-term administration [6]. Their low tolerance often causes poor compliance and thus limited response [7]. Accordingly, the proportion of patients with unsatisfactory response to these earlier treatments is high, especially in patients with migraine at high-frequency of attacks [8].

The Eurolight study, a cross-sectional questionnaire-based survey among headache patients, investigated migraine prevalence and frequency, as well as utilization of medical services and medications in ten European countries including Germany [3, 9, 10]. However, applied therapeutic algorithms, number of failed or successful treatment options and medical need for new treatment options had not been addressed in full detail.

The main objective of the study PANORAMA (**P**atient M**A**nageme**N**t and **O**rganization in mig**RA**ine specialized centers in Ger**MA**ny) was to shed light into the current health care situation of migraine patients in Germany before new targeted treatment options like monoclonal antibodies against CGRP receptor or CGRP ligand became available. Furthermore, the survey aimed to characterize the infrastructure of specialized headache centers and neurologists with a focus on headache.

## Methods

PANORAMA was conducted from October-2017 to October-2018 and collected data from 119 headache and neurology centers in Germany. The project comprised three questionnaires, two out of them were filled out during structured interviews with physicians and one based on an individual database research including patient chart reviews performed by the physicians.

The first interview generated an individual center profile, including size and type of the center, organizational structure, qualification, network structure and role of the nurse. The second questionnaire aimed to characterize the center’s migraine patient cohort. To this end, participating physicians were asked to perform patient chart reviews and to comment on the proportion of episodic and chronic migraine patients and their current and previous medical care. If a prophylactic treatment was insufficient, not well tolerated or discontinued due to various reasons it was considered a treatment failure. Data was not obtained in a controlled setting and source data verification was not performed at this point, thus the provided answers in the second questionnaire might arise from analyses of patient chart reviews as or solely reflect the opinion of the answering physician. The third questionnaire was used to collect information about qualitative aspects of the migraine healthcare situation, including (1) the classification of chronic or episodic migraine along with factors to determine migraine severity (data not shown), (2) implementation of therapy recommendations in daily practice (data not shown) and (3) expectations regarding novel prophylactic migraine therapies under development in respect to e.g. efficacy or safety.

In general, collected data summarized using descriptive statistics. No data validation or data imputation was performed. Answers could comprise absolute numbers or percentages. If indicated, percentages were also calculated from absolute numbers, but not vice versa. Numbers shown have been assessed on site individual level of each participating center. If questions allowed free-text answers, these were categorized by the use of keywords and subsequently summarized.

## Results

**Fig. 1:**
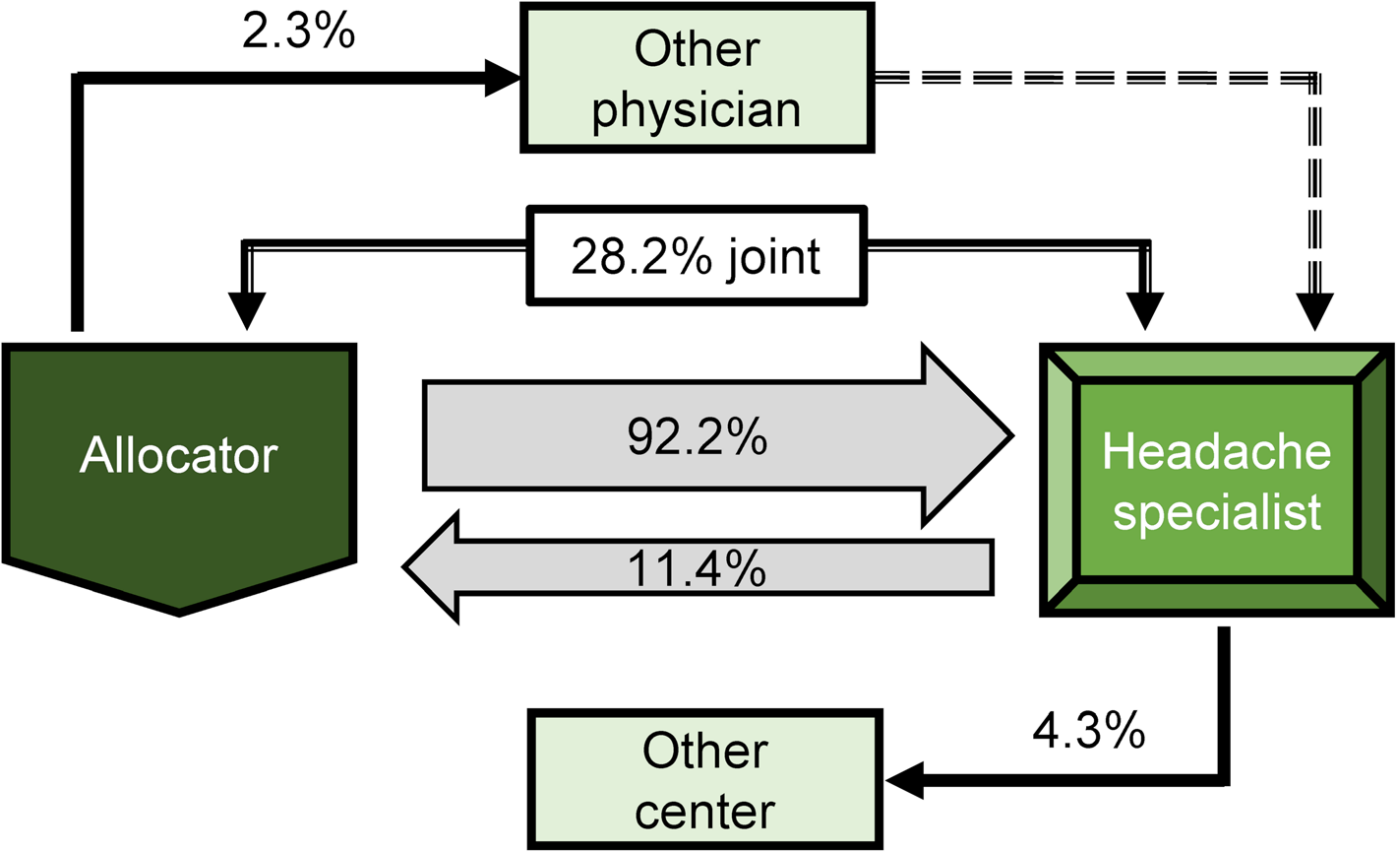
Schematic overview of the referral structure of medical care for migraine patients

The analysis of the first interview characterized the individual center profiles. The survey revealed that in 2017/2018, 92.2 % of the patients treated by a headache specialist have been referred by a healthcare professional and only 11.4 % of these patients were then referred back to their allocator or another center. For more than a quarter (28.2 %) of the migraine patients medical care was provided by joint treatment by the allocating physician and the headache specialist (Fig. 1).

**Fig. 2:**
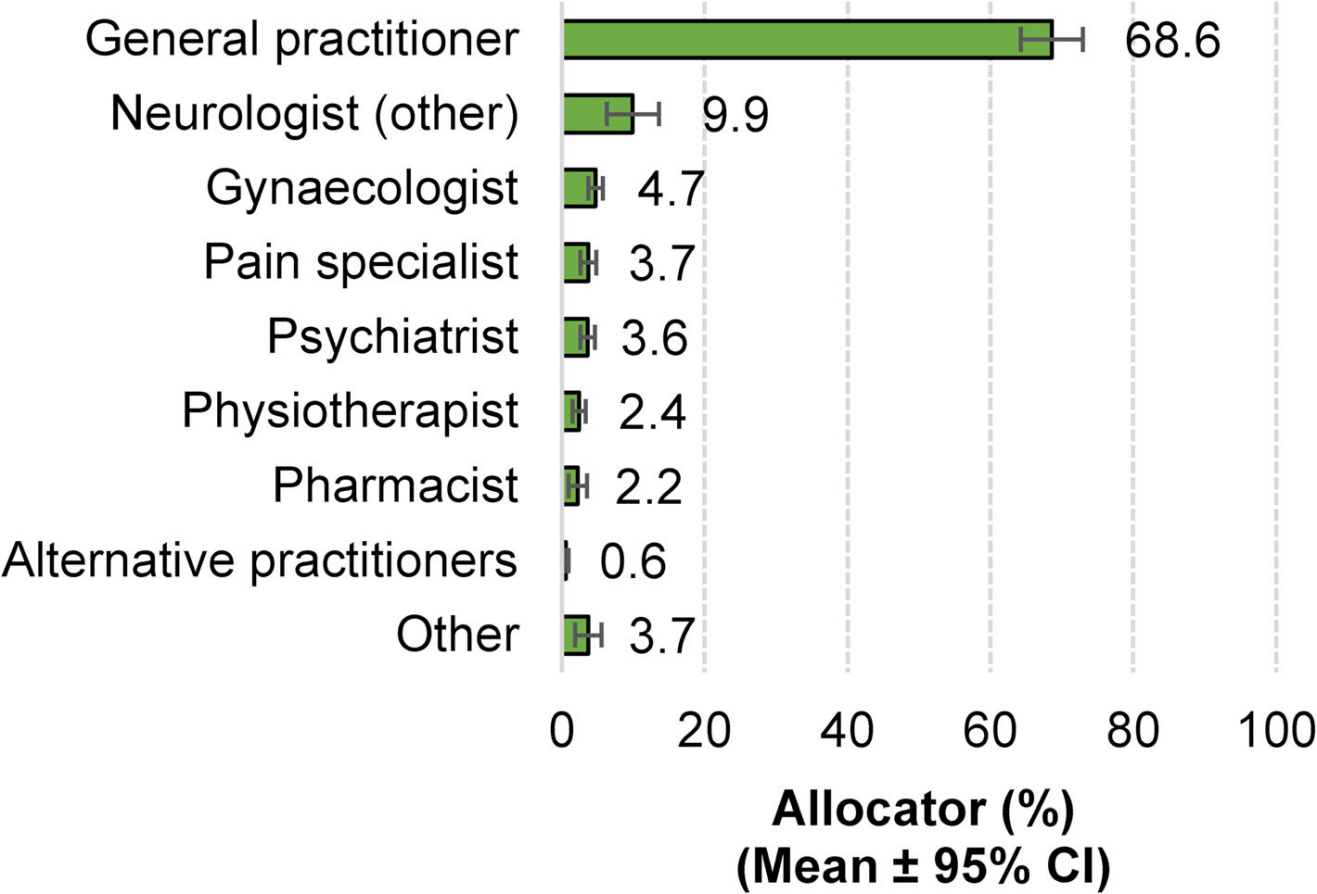
Referral of patients to headache specialists by type of physician or health care professional

More than two thirds of the patients (68.6 %) that were referred to headache specialists were referred by a general practitioner and every tenth patient (9.9 %) by another neurologist. Referral by other health care professionals account for only 20.9 % of the remaining referrals (Fig. 2).

Altogether, 41.6 % of the migraine patients at headache specialists were receiving a prophylactic migraine treatment during that time (Fig. S1, supplementary information). 17.9 % of their patients had discontinued and stopped treatment with a migraine prophylaxis.

**Fig. 3:**
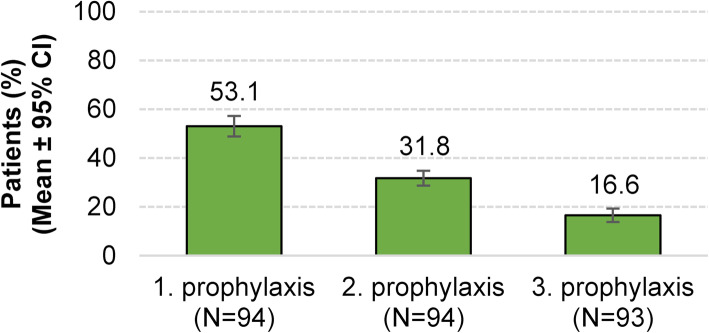
Proportion of patients at distinct stages of prophylaxis at specialized centers in 2017/2018 (*n* = number of questionnaires included in the evaluation)

**Fig. 4:**
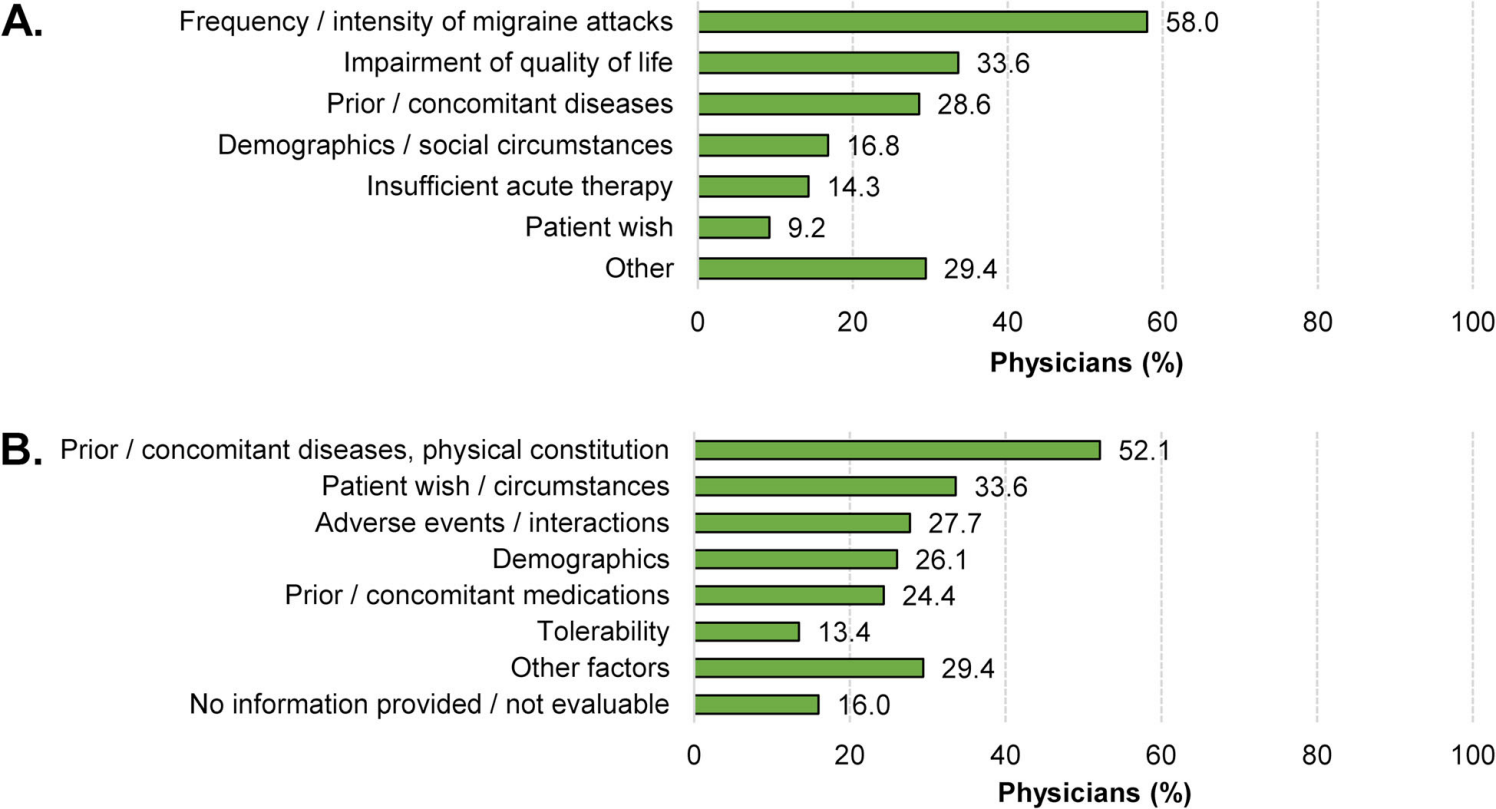
[**A**] Reasons for prophylactic migraine treatment initiation (multiple answers allowed); [**B**] Reasons for prophylactic migraine treatment choice (multiple answers allowed) in 2017/2018

About half (53.1 %) of the patients receiving a prophylactic treatment were on their first medication, one third (31.8 %) on their second medication, and one sixth (16.6 %) on their third prophylactic migraine treatment (Fig. 3). In Germany, preventive migraine therapy is initiated as medical monotherapy only.More than half of the physicians (58.0 %) considered frequency and intensity of migraine attacks as the most important factors influencing their decision to initiate prophylactic migraine treatment. Impairment of quality of life (33.6 %) and prior or concomitant diseases (28.6) were also considered as important by some of the physicians. Other factors like the patient wish or an insufficient response to acute treatment seem to play subordinate roles (Fig. 4 A).

For the choice among the established prophylactic migraine treatments in 2017/2018, more than half (52.1 %) of the physicians considered prior or concomitant diseases and the physical constitution of the patient as key factors. However, about one quarter to one third of the physicians stated also patient preferences and patient circumstances (33.6 %), adverse events and interactions (27.7 %), demographics (26.1 %) and prior or concomitant medications (24.4 %) as important factors for their choice of a particular prophylactic treatment (Fig. 4B).

**Fig. 5:**
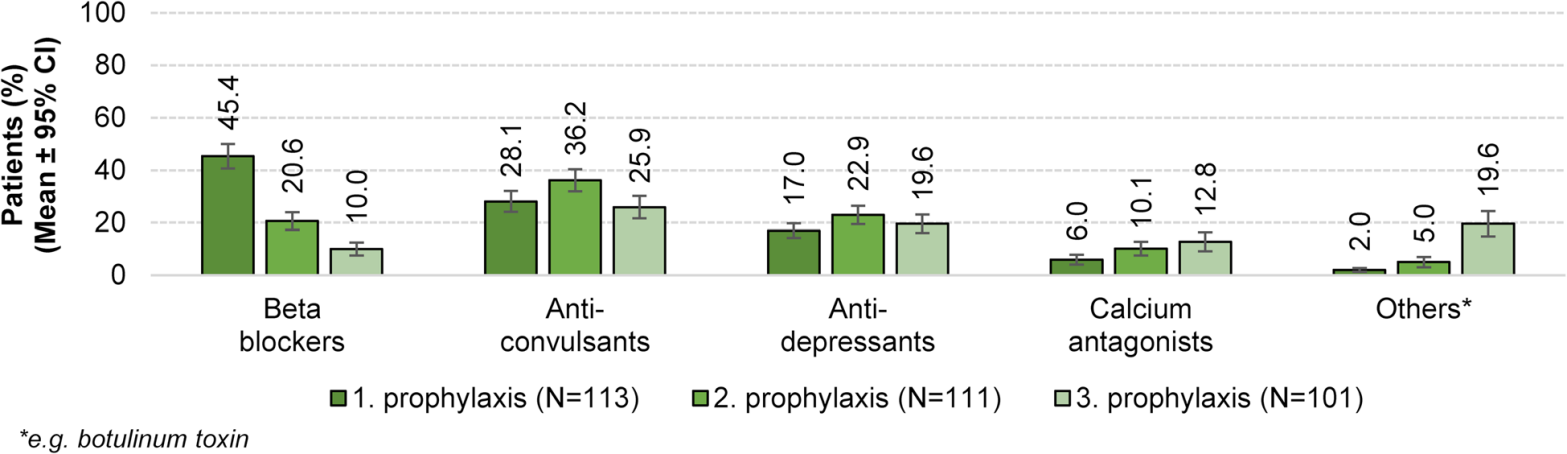
Treatment sequence of prophylactic migraine substance classes (predefined answers, *n* = number of questionnaires included in the evaluation)

Overall, most of the patients received beta-blockers (45.5 %), while 28.1 % received anticonvulsants and 17.0 % antidepressants as first prophylactic treatment. If the first prophylactic treatment was a so-called treatment failure, these particular medications were used in different sequences as second or third prophylactic treatment. With increasing number of prophylactic treatment failures, the proportion of patients receiving calcium antagonists increased approximately 2-fold from first (6.0 %) to third prophylactic treatment (12.8 %). In line with this, the proportion of patients receiving other prophylactic treatments, like botulinum toxin increased substantially with increasing number of prophylactic treatment failures and was almost ten-fold higher after failure of the third prophylactic treatment compared to end of the first prophylactic option (2.0 % vs. 19.6 %) (Fig. 5).

**Fig. 6:**
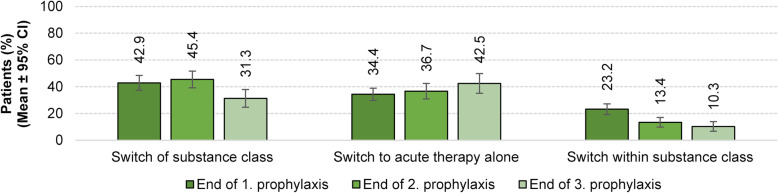
Therapeutic options after discontinuation of prophylactic migraine treatment

Upon discontinuation of the first migraine prophylaxis, 42.9 % of the patients switched substance class, 34.4 % switched to acute therapy alone and only 23.2 % switched to another drug within substance class. The more prophylactic treatments fail, the lower the proportion of patients receiving drugs of the same substance class. Compared to first prophylactic treatment, there was a more than 50 % decrease in the proportion of patients receiving a different drug from a certain substance class after discontinuation of third prophylactic treatment (23.2 % vs. 10.3 %). Conversely, the proportion of patients switching acute therapy alone steadily increased with increasing number of prophylactic migraine treatment failures, i.e. from 34.4 % after end of first, up to 42.5 % after end of third prophylactic migraine treatment. Summarizing, whereas after discontinuation of first prophylactic migraine treatment, switch of substance class seems to be the method of choice, it is rather switching to acute therapy after discontinuing the third prophylactic treatment (Fig. 6).

The majority of the surveyed physicians considered prophylactic treatment options available before monoclonal antibodies targeting the CGRP pathway came onto the market, as not sufficient. In this regard in 2017/2018, over 90.7 % of the participating physicians stated a high to very high need for novel prophylactic migraine treatments including monoclonal antibodies (Fig. S2, supplementary information).

## Discussion

In the course of the PANORAMA study, a snapshot of the German medical and health care situation of migraine patients in 2017/2018 was shown, uncovering areas with need for improvement. The data draws a picture of a patient journey through referral structures and different treatment sequences that was characterized by different and inconsistent treatment algorithms. Both the type and sequence of particular earlier prophylactic migraine treatments varied as well as a common rule to e.g. switch substance class or switch within a substance class. The high proportion of patients only depending on acute treatment, further increasing with number of treatment failures, additionally highlights a lack of suitable preventive therapeutic options at that time. This and the probably increasing frustration of patients mirror the claim for novel migraine therapies, made by the physicians. The development and approval of a new therapeutic approach targeting the CGRP pathway by monoclonal antibodies, as well as the development of novel biological prophylactic migraine treatments [11–17] with increased efficacy, tolerability and safety hold the promise of overcoming former therapy drawbacks. In this context, a large controlled clinical trial investigating erenumab in patients with episodic migraine showed its efficacy in reducing the number of monthly migraine days and use of acute medication, as well as an improvement in Physical-Impairment- and Everyday-Activities Scores [18].

The results also displayed that most patients are referred to the headache specialist by a general practitioner. However, a considerable proportion of migraine patients are referred by another neurologist. Although the reason behind this is not clear, it might be speculated that these patients were either referred to hospital’s outpatient departments for clinical study purposes or for a specific kind of migraine treatment. This would support the assumption of an inadequate migraine prophylaxis at that time along with the wish of the patients to receive a more targeted, migraine specific therapy, be it pharmacological or non-pharmacological.

Furthermore, PANORAMA revealed still an unmet need for an improved care infrastructure for migraine patients that would enable proper access to appropriate therapies. By the time the Eurolight project started in autumn 2008, less than 20 % of patients at headache specialists in Germany received preventive migraine treatments [9]. About ten years later, data from PANORAMA suggest that the proportion of patients receiving prophylactic migraine treatment at headache specialists in Germany has increased (> 40 % according to Figure S1). Conversely, about 60 % of migraine patients still miss a potentially beneficial prophylactic treatment and may not be treated adequately. Even patients starting prophylactic treatment often discontinued prophylaxis and switched to acute therapy alone, which indicates a need for an effective prophylactic treatment, which was not available in 2017/2018.

Results and interpretations deduced from this data collection are limited by the reduced sample size of the survey and the subjective answering of the survey by the participating physician.

The medical health care landscape for migraine patients is complex. Besides the need for novel prophylactic treatment options and an improved health care infrastructure, other factors influencing health utilization patterns should be considered. In this context, a recent study published by Müller et al. revealed that consultation of a headache specialist in Germany also depends on different factors including number of headache days, Headache Impact Test Score and employment status of a patient. Especially for the latter aspect, the survey suggested a significant lower consultation rate of self-employed persons  [19].

Thus, the availability of novel prophylactic treatments, like CGRP-targeted therapies, and improvement of medical care of migraine patients elucidated by this study, are suggestive of an overall improvement of the health care situation of migraine patients in Germany over the last decade. However, efforts have still to be maintained and more real world data on the new treatment paradigm have to be collected in order to ensure and improve patients’ access to their optimal treatment.

### Conclusions

PANORAMA gave a comprehensive overview of the migraine healthcare landscape data in Germany in 2017/2018, elucidates a lack of common treatment algorithms and reveals a high demand for defined therapy strategies. The data may also serve as a basis for a better comparison and classification of future improvements in the health care situation in Germany resulting from newly approved migraine-specific preventive medications.

## Supplementary Information

**Additional file 1:**

## Data Availability

The datasets used and/or analyzed during the current study are available from the corresponding author on reasonable request.

## Abbreviations

CGRP: Calcitonin Gene-Related Peptide
QoL: Quality of life
