# Supplementary material for: Migraine patients in Germany - need for medical recognition and new preventive treatments: results from the PANORAMA survey
Source: J Headache Pain. 2021 Sep 9;22(1):106. doi: 10.1186/s10194-021-01316-5 (PMC8428142; doi:10.1186/s10194-021-01316-5)
Supplement: Supplementary file 1 — Additional file 1: [file 10194_2021_1316_MOESM1_ESM.pdf]

Supplementary information

Fig. S1

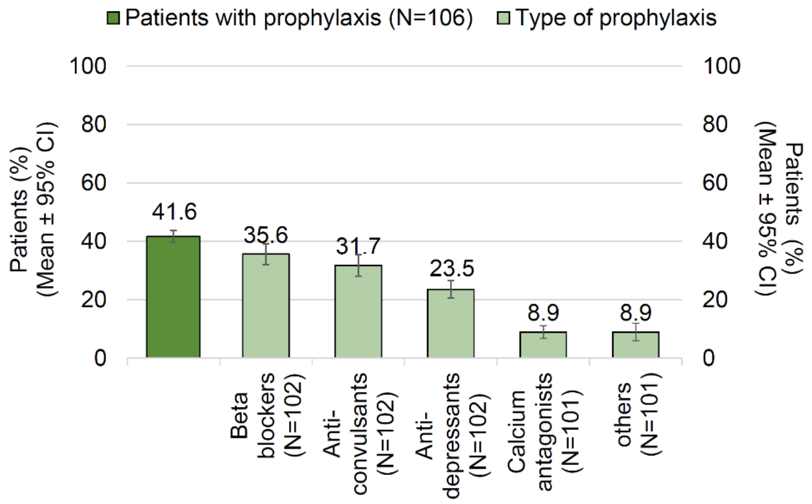

Proportion of patients with prophylaxis treated by headache specialists and type of used prophylactic migraine treatment. 41.6% of the patients treated by a headache specialist receive a prophylactic migraine treatment, most of them receiving beta blockers (35.6%), anticonvulsants (31.7%) and antidepressants (23.5%), (predefined answers)

Fig. S2

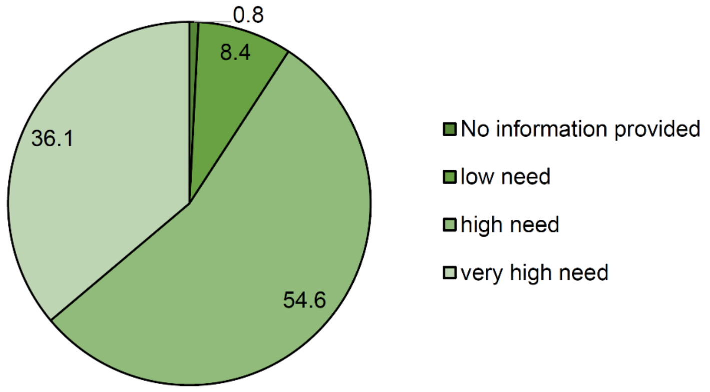

Physicians' assessment of medical need for novel prophylactic migraine treatments. Of the surveyed physicians, 36.1% see a very high, 54.6% a high and 8.4% no need for novel (biological) prophylactic migraine treatments.
